# Carveol a Naturally-Derived Potent and Emerging Nrf2 Activator Protects Against Acetaminophen-Induced Hepatotoxicity

**DOI:** 10.3389/fphar.2020.621538

**Published:** 2021-01-28

**Authors:** Zaif Ur Rahman, Lina Tariq Al Kury, Abdullah Alattar, Zhen Tan, Reem Alshaman, Imran Malik, Haroon Badshah, Zia Uddin, Atif Ali Khan Khalil, Naveed Muhammad, Saifullah Khan, Amjad Ali, Fawad Ali Shah, Jing Bo Li, Shupeng Li

**Affiliations:** ^1^ Shenzhen University Clinical Research Center for Neurological Diseases, Health Management Center, Shenzhen University General Hospital, Shenzhen University Clinical Medical Academy, Shenzhen University, Shenzhen, China; ^2^ Department of Pharmacy, Abdul Wali Khan University, Khyber Pakhtunkhwa, Pakistan; ^3^ College of Natural and Health Sciences, Zayed University, Abu Dhabi, United Arab Emirates; ^4^ Department of Pharmacology and Toxicology, Faculty of Pharmacy, University of Tabuk, Tabuk, Saudi Arabia; ^5^ Riphah Institute of Pharmaceutical Sciences, Riphah International University, Islamabad, Pakistan; ^6^ Department of Pharmacy, COMSATS University Islamabad, Abbottabad Campus, Abbottabad, Pakistan; ^7^ Department of Biological Sciences, National University of Medical Sciences, Rawalpindi, Pakistan; ^8^ Department of Microbiology and Biotechnology, Abasyn University Peshawar, Khyber Pakhtunkhwa, Pakistan; ^9^ Department of Botany, University of Malakand, Khyber Pakhtunkhwa, Pakistan; ^10^ State Key Laboratory of Oncogenomics, School of Chemical Biology and Biotechnology, Shenzhen Graduate School, Peking University, Shenzhen, China

**Keywords:** acetaminophen, carveol, hepatotoxicity, anti-inflammatory, Nrf2 pathway

## Abstract

Acetaminophen (N-acetyl p-aminophenol or APAP) is used worldwide for its antipyretic and anti-inflammatory potential. However, APAP overdose sometimes causes severe liver damage. In this study, we elucidated the protective effects of carveol in liver injury, using molecular and *in silico* approaches. Male BALB/c mice were divided into two experimental cohorts, to identify the best dose and to further assess the role of carveol in the nuclear factor E2-related factor; nuclear factor erythroid 2; p45-related factor 2 (Nrf2) pathway. The results demonstrated that carveol significantly modulated the detrimental effects of APAP by boosting endogenous antioxidant mechanisms, such as nuclear translocation of Nrf2 gene, a master regulator of the downstream antioxidant machinery. Furthermore, an inhibitor of Nrf2, called all-trans retinoic acid (ATRA), was used, which exaggerated APAP toxicity, in addition to abrogating the protective effects of carveol; this effect was accompanied by overexpression of inflammatory mediators and liver = 2ltoxicity biomarkers. To further support our notion, we performed virtual docking of carveol with Nrf2-keap1 target, and the resultant drug-protein interactions validated the *in vivo* findings. Together, our findings suggest that carveol could activate the endogenous master antioxidant Nrf2, which further regulates the expression of downstream antioxidants, eventually ameliorating the APAP-induced inflammation and oxidative stress.

## Introduction

Liver diseases are associated with an increased number of disability cases, and approximately 50 million liver-associated morbidity and mortality reports are documented each year (Gorrell, 2005). Because of the persistent and continuous involvement of the liver in metabolic processes, the accumulation of free radicals overwhelms the natural defense system, making the liver one of the most vulnerable organ. These free radicals are the prominent cause of oxidative stress-induced thiol depletion and lipid peroxidation, which subsequently trigger toxic cascading events and eventually lead to various hepatic pathologies, such as cirrhosis and acute or chronic hepatitis (Abo-Haded et al., 2017).

Paracetamol (acetaminophen, N-acetyl p-aminophenol, or APAP) is a non-prescription drug with both analgesic and antipyretic activities, and this drug is included in several preparations, either as a single moiety or in combination (Ghaffar and Tadvi, 2014). It has a large therapeutic window and is generally safe; however, when abused in large doses, it leads to severe hepatic necrosis and hepatic failure (Al-Fartosi et al., 2011). Currently, paracetamol is considered the chief causative agent of acute liver failure due to accidental overdose, which requires a liver transplant in some extreme cases (Gopal et al., 2011; Du et al., 2016). At a high dose, APAP is metabolized to glucuronic acid or sulfate conjugates (Sharma et al., 2016) and is transformed into the pro-reactive cytotoxic intermediate N-acetyl-phenzoquinoneimine (NAPQI), which is responsible for oxidative stress and intracellular glutathione (GSH) depletion (Shah et al., 2014; Hellerbrand et al., 2017; Zai et al., 2018). The covalent binding of NAPQI to mitochondria initiates cascading pathological processes, such as free radical formation and peroxynitrite accumulation, further supplemented by the release of pro-inflammatory cytokines and mediators, all of which collectively exacerbate acute and chronic liver necrosis (Dalaklioglu et al., 2013; Hennig et al., 2018). Various hepatoprotective strategies have been evaluated to protect the liver from toxins. The current clinical practice recommends silymarin as a hepatoprotective supplement to cope with various liver insults, including APAP and methotrexate (MTX) (Crowell et al., 1992). The transcription factor Nrf2 is an integral part of the host cellular defense mechanism against oxidative stress and electrophilic insult. Nrf2 binds to antioxidant response elements (ARE) at the promoter site, which in turn encodes several antioxidant/phase-II detoxifying enzymes and other relevant stress-responding factors (Thompson Coon and Ernst, 2002). Previous studies have revealed the involvement of Nrf2-ARE signaling in attenuating inflammation in several pathologies, such as stroke, and other disorders (Ma and He, 2012; Ning et al., 2018). Hence, dysregulation of Nrf2 signaling results in increased susceptibility to oxidative stress and inflammatory damage. Previous studies have demonstrated that Nrf2 plays a critical role in the regulation of inflammation and oxidative stress, which are linked to the pathophysiologies of several diseases. Therefore, Nrf2 can be considered a potential pharmacological target to be investigated against various insults, including APAP.

**Figure 1:**
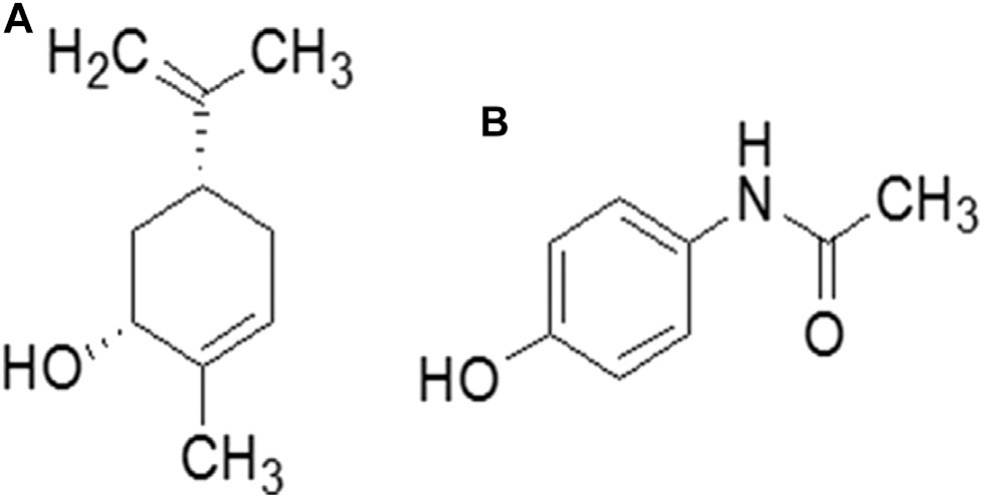
Chemical structure of **(A)** carveol and **(B)** paracetamol.

Natural drug moieties are an attractive source of new drugs, owing to their rich antioxidant potential. Several natural drugs have hepatoprotective potential against a variety of mediators, including free radicals and inflammatory factors (James et al., 2003; Lee et al., 2006). Carveol, a monoterpene phenol, is isolated from the essential oils extracted from the plant family *Lamiaceae* or *Labiatae*, which includes the genera *Thymbra*, *Origanum*, *Corydothymus*, *Satureja*, and *Thymus* (Figure 1) (Pastore et al., 2003). It has long been used in traditional Chinese medicine as an antispasmodic, a carminative, and an astringent (Aleksunes et al., 2008) and has been evaluated in the treatment of indigestion and dyspepsia (Guo and White, 2016). Carveol has been demonstrated to have antioxidative, antihyperlipidemic, and anti-inflammatory activities, and ameliorate liver toxicity in a mouse model of carbon tetrachloride (Patterson et al., 2013). However, to date, the potential hepatoprotective effects of carveol against APAP have not been evaluated. Taking into consideration the pharmacological value of essential oils extracted from plant sources and the significance of new drug discovery, the current study sought to investigate whether carveol mitigates APAP-induced detrimental effects. If so, the potential underlying molecular and cellular mechanisms should be further delineated to explain the effects of carveol on hepatocellular protection. This will not only expand the understanding of the molecular cascading mechanism of cell death but will also provide some clues to unveil the therapeutic potential of carveol.

## Materials and Methods

### Chemicals and Reagents

The pharmaceutical drugs (silymarin and N-acetyl para aminophenol (paracetamol CAS: 103-90-2, C8H9NO2: APAP) of HPLC grade (99%) were supplied by a local pharmaceutical manufacturer and used as raw material. All the antibodies were procured from Santa Cruz Biotechnology, United States, or Abcam, United Kingdom. Phosphate-buffered saline (PBS) tablets were used for all morphological analyses or fresh buffers were prepared for each use. The details and corresponding catalog numbers of the primary antibodies are HO-1 (SC-13691), TRX (SC-20146), Nrf2 (SC-722), COX-2 (SC-514489), p-JNK (SC-6254), TNF-α (SC-52B83), and p-NFκB (SC-271908). Other immunohistochemistry-related consumables, such as AB and C Elite kit (two vials-SC-2018) and 3,3-diaminobenzidine (DAB) (SC-216567), were also provided by Santa Cruz Biotechnology, United States. The biotin secondary antibody (ab-6789) and DPX mounting media were purchased from Abcam United Kingdom. The p-NFκB enzyme-linked immunosorbent assay (ELISA) kit (Cat # SU-B28069) and Nrf2 kit (cat. no. SU-B30429) were purchased from Shanghai Yuchun Biotechnology, China. HO-1 (cat. No. E-EL-R0488), and TNF-α (E-EL-R0019) ELISA kits were purchased from Elabscience.

### Animals and Drug Treatment

Male BLAB/c mice, weighing 30–35 g and 8–10 weeks old, were housed (three per cage) at the facility of the Riphah Institute of Pharmaceutical Sciences (RIPS), under-documented protocols (temperature: 22 ± 1 °C; humidity: 50 ± 10%). Strict laboratory protocols were followed during all experimental procedures. The animals were kept for some days at the facility before the experimental procedures, and the body weights were constantly checked every day throughout the study. Furthermore, we strictly followed the approved protocols and guidelines of the institutional research ethical committee (REC) of the Riphah Institute of Pharmaceutical Sciences (RIPS), Islamabad (Approval ID: Ref. No. REC/RIPS/2018-19/A202), which are similar to the ARRIVE guidelines, with some minor exemptions. We adopted the human endpoint criteria for euthanizing the mice if they displayed a severe sign of distress or suffering. The mice were subjected to the following experimental protocols. Two separate cohorts of animals were used for the experiments as follows:

### Experimental Cohort 1

**Figure 2:**
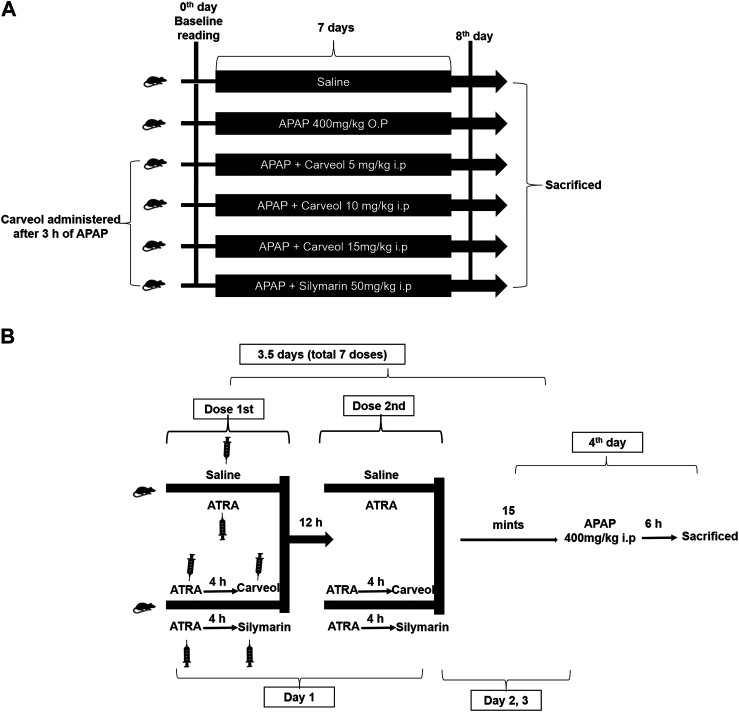
Experimental design 1 **(A)**. Carveol, APAP, and silymarin were prepared in 0.9% NaCl, while ATRA was prepared in a mixture of 2% dimethyl sulfoxide and 0.9% NaCl (*n* = 10/group). Baseline reading was recorded at the beginning (0th day of the experiment), and mean weight and food intake were monitored during the entire experiment **(B)** Diagrammatic illustration of *in vivo* study for the Nrf2 signaling pathway. The treatment protocol was continued for 3.5 days. In all these groups, a single loading dose of paracetamol was injected (400 mg/kg, i. p.) 15 min after the last dose of ATRA or carveol or silymarin, except in the saline group. Serum samples and liver tissues were collected after 6 h of the last dose.

The mice treatment protocol is indicated in Figure 2A, and the following groups were employed. 1) Saline group: the mice received saline (0.9% NaCl) injection intraperitoneally (i. p.) for seven consecutive days, 2) APAP group: the mice received a single dose of paracetamol orally/per os (400 mg/kg, p. o.) for 7 days, 3) Three different groups of APAP + carveol: the mice received paracetamol orally for seven consecutive days, followed by a single i. p. injection of different doses of carveol, namely APAP + carveol 5 mg (5 mg/kg, i. p.); APAP + carveol 10 mg (10 mg/kg, i. p.); APAP + carveol 15 mg (15 mg/kg, i. p.), 4) APAP + silymarin group: the mice received paracetamol orally for seven consecutive days, followed by silymarin (50 mg/kg, i.p). Carveol, APAP, and silymarin were prepared in 0.9% NaCl (*n* = 14 per/group). At the end of the experiment, the mice were anesthetized with xylazine and ketamine (i. p.); blood was collected via cardiac puncture and was processed for biochemical analysis. Samples from the liver were taken and frozen at −50 °C or preserved in 4% paraformaldehyde for ELISA or paraffin sectioning, respectively. Next, 4 μm thin hepatic tissue sections were made with a rotary microtome from the paraffin block, for histological analysis. Overall, three mice died during the experimental procedures, two from APAP and one from the Carveol + APAP groups, which we excluded from the experiment. The saline-treated mice survived throughout the experiments.

### Experimental Cohort 2

The mice treatment protocol is indicated in Figure 2B, and the following groups were employed with *n* = 10/gp. 1) Saline group: the mice received saline (i. p.), 2) APAP + ATRA group: the mice received seven injections of ATRA (10 mg/kg, i. p.) at 12 h intervals, 3) APAP + ATRA + Carveol group: the mice received carveol 15 mg/kg i.p 4 h after each ATRA dose, and 4) APAP + ATRA + silymarin group: the mice received silymarin 50 mg/kg i.p 4 h after each ATRA dose. For all the groups except saline, APAP 400 mg/kg was administered (i. p.) 15 min after the last dose of ATRA (APAP + ATRA group) or carveol (APAP + ATRA + carveol) or silymarin (APAP + ATRA + silymarin). At the end of the treatment, the mice were sacrificed and samples were collected.

### Hematoxylin and Eosin (H and E) Staining

Hematoxylin and eosin staining was performed according to our previous protocols (Ali et al., 2019; Ansari et al., 2019; Ullah et al., 2020). Briefly, the non-coated slides initially underwent a deparaffinization step with xylene, followed by hydration (graded ethanolic series), and finally with water. The slides were incubated in a Coplin jar containing hematoxylin to stain the nucleus. The slides were washed with water and traced for nuclear staining, using a compound microscope. The slides were then dipped in 1% HCl, 1% ammonia water, and rinsed with water. Eosin staining was then performed, the slides were subjected to dehydration, fixed in xylene, and covered with coverslips. Five images per slide were obtained using a light microscope (Olympus, Japan) at the same threshold intensity, and were later analyzed using ImageJ software to quantify the number of distorted, vacuolated, infiltrated, and surviving cells.

### Liver Functional Biomarkers

Enzymes, such as AST, ALT, phosphatases, and total bilirubin (TB), were spectroscopically analyzed using an autoanalyzer (Olympus AU-2700) according to the manufacturer’s instructions.

### Oxidative Enzyme Analysis and Lipid Peroxidation

The non-enzymatic glutathione (GSH) level and the enzymatic glutathione S-transferase (GST) activity were determined as previously described (Ullah et al., 2020). After homogenizing liver tissue samples (cohort 1), the supernatant was collected. For the assay, the stock of 0.2 M sodium phosphate buffer was prepared as Na_2_HPO_4_.2H_2_O and NaH_2_PO_4_ (pH 8). For sample loading, buffer (153 μL), freshly prepared 1 mmol DTNB (40 μL), and the supernatant (6.6 μL) were sequentially mixed, and after 15 min, the absorbance of this mixture was determined using a spectrophotometer at 412 nm. A mixture of DTNB solution and phosphate buffer served as the control, whereas the buffer was used as a blank. The absorbance was calculated from the values of the absorbance of the control and the sample and expressed as µmol/mg protein. To detect GST activity (Imran et al., 2020), the stock of 0.1 M potassium phosphate buffer was prepared as KHPO_4_ and KH_2_PO_4_ (at a 1:2 ratio, pH 6.5). For the assay, 1 mmol GST and 1 mmol CDNB were also prepared; then GST solution, CDNB, potassium buffer, and tissue homogenate were mixed (at a 1:1:27:1 ratio) and the optical density was determined at 340 nm. The phosphate buffer was used as a blank, and the assay mixture without homogenate was used as a control. GST activity was calculated using the extinction coefficient of the product and expressed as nmoles of CDNB conjugated/min/mg protein.

### Determination of Lipid Peroxidation (LPO) in Tissue

Oxidative stress augmented the oxidation of macromolecules, such as lipids, which can be quantified by the thiobarbituric acid reactive substance (TBARS) levels. A previously reported protocol was adopted for the LPO assay, with minor changes (Imran et al., 2020). Approximately 40 µL of tissue supernatant was added to a freshly prepared solution of ferric ammonium sulfate and incubated for some time. Next, after the addition of 75 μL of thiobarbituric acid (TBA) to the mixture, the color changed, and the absorbance of the resultant mixture was immediately measured using a microplate reader (at 532 nm). The levels were expressed as nmol Tbras/min/mg protein (Iqbal et al., 2020).

### Immunohistochemical Analysis

We used coated slides for immunohistochemical studies as described in a previous study (Rana et al., 2020). The slides were subjected to deparaffinization and hydration protocols as discussed for H and E. To unlock the antigenic epitopes from paraformaldehyde, proteinase K was applied to the tissue, followed by PBS rinsing. After hydration, the slides were not allowed to dry at any stage of the immunohistochemical analysis. Before blocking with normal goat serum the slides were treated with H_2_O_2_ to eradicate peroxidase activity. The selectivity of serum depends upon the source of the secondary antibody. After blocking for an appropriate time, primary antibodies, such as nuclear factor-κB (p-NFκB), COX2, c-Jun N-terminal kinase (p-JNK), HO-1, TNF-α, and Nrf2 (dilution 1:100, Santa Cruz Biotechnology), were applied overnight in a moistened box in the refrigerator. The following morning, the slides were removed and kept for 1 h at room temperature in a moistened chamber. After rinsing with PBS, the biotinylated secondary antibody (goat anti-mouse and goat anti-rabbit) was applied, followed by the application of ABC reagent (SCBT United States) in a humidified chamber, and the slides were rinsed with PBS and stained with DAB. The slides were then dried, dehydrated in ascending ethanolic series, fixed in xylene, and covered with coverslips. The liver positive cells for all the primary antibodies were quantified using the ImageJ software.

### Enzyme-Linked Immunosorbent Assay (ELISA)

p-NFκB, HO-1, Nrf2, and TNF-α levels were quantified using an ELISA kit as per the manufacturer’s instructions (for detailed chemicals and reagents). Approximately 50 mg of the tissue sample was homogenized, using PBS (also containing PMSF as a serine inhibitor), at 15,000 RPM, followed by centrifugation and collection of the supernatant. Protein concentration in each homogenate was calculated using the BCA kit (Thermo Fisher), and the resultant protein concentration was added to each well to determine the level of the respective proteins, using an ELISA reader. The resultant picograms of cytokines per milliliter (pg/ml) were then converted pg/mg total protein).

### Real-Time Polymerase Chain Reaction (RT-PCR)

Total RNA was extracted from the freshly isolated liver of mice in experimental duplicates using the TRIzol method. 20 µL of M-MuLV reverse transcriptase was used to dilute 1 μg of RNA and used this mix to synthesize cDNA with a cDNA synthesis kit (vivantis cDSK01-050 Sdn. Bhd, Malaysia). To estimate the gene expression of Nrf2 quantitatively, real-time PCR was performed using the 2X HOT SYBR Green qPCR master mix (Solar Bio cat # SR1110) and real-time Mic PCR (BioMolecular System) according to the manufacturer specifications. The sequence of the primers used for amplification was Nrf2 Forward: CCA​TTT​ACG​GAG​ACC​CAC​CGC​CTG and Reverse: CTC​GTG​TGA​GAT​GAG​CCT​CTA​AGC​GG and GAPDH, Forward: AGG​TCG​GTG​TGA​ACG​GAT​TTG and Reverse: TGT​AGA​CCA​TGT​AGT​TGA​GGT​CA (Wakabayashi et al., 2014). The relative gene expressions of Nrf2 was determined by the 2 ^−ΔΔCT method for real-time quantitative PCR.

### Bioinformatic Studies

*In silico* studies were performed as previously described (Shah and Rashid, 2020). Briefly, the 3-dimensional structures of cyclooxygenase (COX2) PDB ID: IPXX, interleukin (IL-1β) PDB ID: 2MIB, PDB ID: 2TNF for TNF-α, PDB ID: 3TTI for JNK, PDB ID: ILE5 for nuclear factor-kB (NFκB), PDB ID: 1DVE for HO-1, and PDB ID: 2LZ1 for Nrf2 were downloaded from the RCSB protein data bank in Discovery Studio (DSV). Docking studies require PDB and mol2 format, for which the 3D structure of the proteins and ligand carveol were downloaded in the respective format. Both protein and ligand were loaded into the PyRx docking software, and drug-receptor interactions were evaluated by binding the energy values (E-value). The E-values further validated the best pose of the ligand in the complex, and by DSV, the best orientation and interaction were prepared and analyzed. The structure of carveol was collected from online sources.

### Statistical Analysis

The symbols ∗, #, $, and β were used to indicate a significant difference. * or β indicates differences compared to saline, while # to APAP and $ represents a significant difference to APAP + ATRA. All the data are expressed as mean ± SEM, and ImageJ software was used to analyze all the histological data (ImageJ 1.30; https://imagej.nih.gov/ij/). Bodyweight and food intake data were analyzed using a repeated two-way ANOVA, and the remaining data were analyzed using one-way ANOVA with Tukey’s multiple comparison test as a post-hoc test.

## Results

### Results of Experimental Design one

#### Effect of Carveol on Body Weight and Food Intake

**Figure 3:**
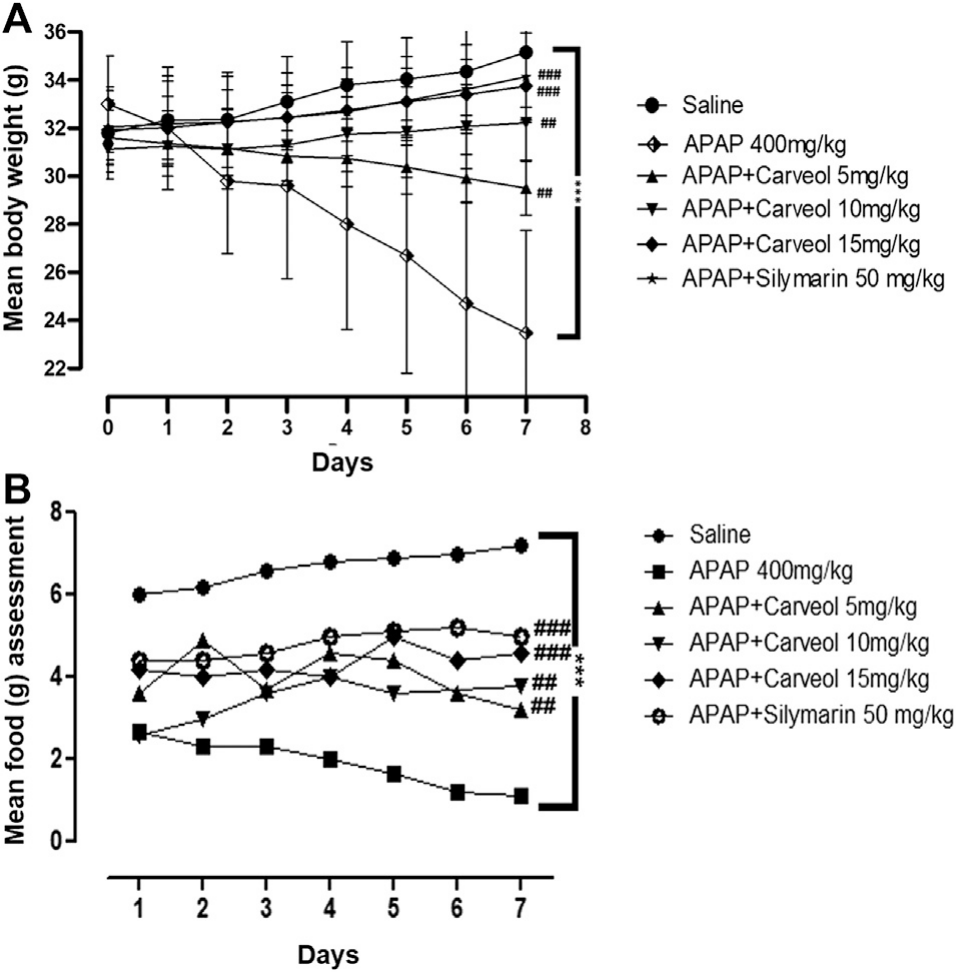
Effect of carveol treatment on physical parameters, such as body weight and food intake. **(A)** Bodyweight changes **(B)** Mean food intake. The number of animals was 14/group, and the results were analyzed using a repeated two-way ANOVA test. ###*p* < 0.001, ##*p* < 0.01 compared to the APAP group, ***p < 0.001, compared to the saline group.

Our results showed that APAP induced a dramatic and persistent loss of body weight. However, carveol prevented body weight loss in a dose-dependent manner (Figure 3A). Carveol, at 15 mg/kg per day, showed the most significant effect on body weight (*p* < 0.001). Bodyweight reduction could be attributed to decreased food intake, as APAP-treated mice consumed significantly less amount of food; however, carveol ameliorated this effect in a dose-dependent manner, which is comparable to that of silymarin (50 mg/kg) (Figure 3B, *p* < 0.001).

#### Carveol Improved the Liver Detriments of APAP

Table 1 shows that APAP significantly disturbed the functional markers (*p* < 0.001), while carveol at different doses normalized the values in the APAP-administered group; carveol at 15 mg exhibited similar effects as those of silymarin.

**TABLE 1:** The protective effect of carveol on liver functional enzymes: APAP augmented the serum level of LFTs and attenuated high-density lipoprotein (HDL) level, while carveol, with respect to APAP, mitigated LFT levels and increased HDL levels.

Treatment	ALT (U/L)	AST (U/L)	TP (g/dl)	TB (mg/dl)	ALP (U/L)	Albumin (g/dl)	LDL (mg/dl)	HDL (mg/dl)
Saline	59 ± 8.71	106 ± 13.89	7.8 ± 0.30	0.33 ± 0.18	98.66 ± 15.53	4.93 ± 0.65	39 ± 5.56	78.66 ± 8.02
APAP 400 mg/kg	194.66 ± 6.02***	276.66 ± 27.02***	3.53 ± 0.40***	2.16 ± 0.30***	220.33 ± 49.44***	2.23 ± 0.30***	102.66 ± 13.05***	37.66 ± 2.51***
APAP + Carveol 5 mg/kg	164.66 ± 10.69	269.33 ± 81.5	5.7 ± 0.45^##^	1.23 ± .37^##^	177 ± 11.78	3.5 ± 36	78.66 ± 17.4	41.66 ± 3.05
APAP + Carveol 10 mg/kg	148.33 ± 11.37^#^	258.66 ± 36.67	5.53 ± 1.19^##^	1.03 ± .20^##^	136.66 ± 3.05^##^	4.23 ± .41^#^	64 ± 21^#^	52.66 ± 6.5^#^
APAP + Carveol 15 mg/kg	95.33 ± 7.37^###^	172.33 ± 14.7^##^	6.6 ± 0.55^###^	0.525 ± 0.03^###^	122.66 ± 8.50^###^	4.5 ± 0.36^##^	65.66 ± 17.67^##^	64.66 ± 7.76^##^
APAP + Silymarin 50 mg/kg	81.33 ± 8.50^###^	138.66 ± 14.22^##^	6.16 ± 0.40^###^	0.67 ± 0.29^###^	130 ± 16.70^###^	5.93 ± 0.85^##^	51.33 ± 9.29^##^	52.33 ± 2.08^#^

The data are presented as means ± SEM and were analyzed using one-way ANOVA with n = 7/group. The symbols ∗∗∗ and ### represent p < 0.001 and the symbol ## represents p < 0.01 values of significant differences. The samples were processed from cohort 1.

#### Carveol Alleviated the Liver Metabolic Deficits Induced by APAP

The liver plays a principal role in the synthesis, storage, secretion, and catabolism of proteins, bilirubin, lipoproteins, and lipids, which represent sensitive markers during liver damage. Our results showed a significant increase in the levels of total protein (TP), albumin, and HDL, accompanied by decreased total bilirubin (TB) and low-density lipoprotein levels (Table 1). These results suggest that liver anabolic and catabolic functions were both severely hampered. Moreover, carveol showed a dose-dependent protective effect, which was comparable to the effects of silymarin at a dose of 15 mg/kg. Furthermore, the effect of carveol on HDL levels was more significant (*p* < 0.01) than that of the silymarin-treated group (*p* < 0.05).

#### Effects of Carveol on Antioxidant Enzymes

Table 2 summarizes the effects of carveol on changes in endogenous enzyme activities, following APAP treatment. APAP stimulated GSH depletion (6.30 ± 4.97), and antioxidant enzyme glutathione-S-transferase (GST) (1.78 ± 1.50) in the hepatic tissue (*p* < 0.001). Treatment with carveol at different doses attenuated the downregulation of GSH (62.29 ± 8.82) and GST (14.2 ± 1.43).

**TABLE 2:** Effect of carveol on oxidative enzymes.

Treatment	GSH (μmol/mg protein)	GST (nmoles of CDNS conjugated/min/mg protein)	TBARS (nmoles Tbras/min/mg protien)
Saline	74.88 ± 13.78	25.42 ± 1.30	79.32 ± 0.70
APAP 400 mg/kg	6.30 ± 4.97***	1.78 ± 1.50***	215.58 ± 5.82***
APA + Carveol 5 mg/kg	44.4 ± 9.26^#^	4.67 ± 0.91	192.54 ± 2.80
APA + Carveol 10 mg/kg	54.49 ± 9.98^##^	8.83 ± 1.62^#^	167.78 ± 2.164^#^
APA + Carveol 15 mg/kg	62.29 ± 18.82^###^	14.2 ± 1.43^##^	131.74 ± 0.64^##^
APAP + Silymarin 50 mg/kg	66.68 ± 11.45^###^	18.20 ± 2.31^##^	115.83 ± 2.164^##^

The symbols ∗∗∗ and ### represent p < 0.001, while the symbol ## or # represents p < 0.01 and p < 0.05, n = 7/group. The data are expressed as mean ± SEM and were analyzed using one-way ANOVA followed by Tukey’s multiple comparison test. The samples were processed from cohort 1.

#### Effect of Carveol on LPO

The LPO content in the liver homogenate of the APAP group was increased to 215.58 ± 5.82, compared to that in the saline group (*p* < 0.001, Table 2). Carveol at 15 mg/kg dose significantly (*p* < 0.01, Table 2) attenuated this content (131.74 ± 0.64), an effect that could be matched to that of the silymarin group.

#### Carveol Protected the Liver From APAP-Induced Cellular Damage

**Figure 4:**
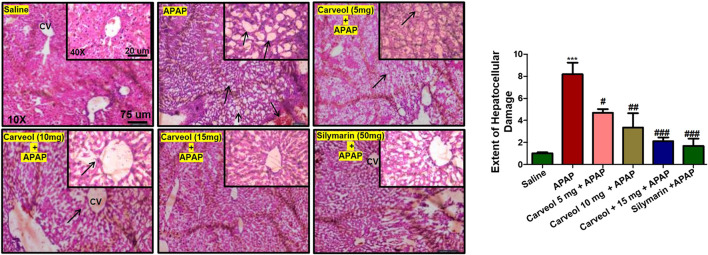
Carveol restored the morphological integrity of the liver, as shown by histological examination. Liver tissue was stained with H and E (magnification, ×10 scale bar 75 μm and 40× scale bar 20 µm). The data are presented as means ± SEM and were analyzed using one-way ANOVA followed by Tukey’s multiple comparison test; *n* = 7/group. The symbols ∗∗∗ and ### represent significant difference values of *p* < 0.001, and ## or # represent significant difference values of *p* < 0.01 or *p* < 0.05 respectively. The slides were processed from cohort 1.

The results of H and E staining revealed significant histopathological changes in the APAP-intoxicated animals (Figure 4, ###*p* < 0.001). Significant alterations were observed in the APAP group, compared to those in the saline-treated animals. The saline group showed normal hepatic cell shape, and there was no vacuolization or lipid globule. Nevertheless, many aberrant morphological features, such as sinusoidal dilatation, hepatocyte degeneration with loss of lobular architecture/hepatocyte disarray, pericentral lymphocytic infiltration, moderate steatosis/fatty degeneration, and abundant inflammatory cell infiltration, were observed in the APAP-treated groups (Table 3). Carveol treatment significantly reversed these histopathological abnormalities induced by APAP, in a dose-dependent manner, as revealed by the microscopic scores (Carveol histopathological score).

**TABLE 3:** Effect of carveol on histopathological scoring.

Groups	Hepatocytes necrosis	Inflammatory cells infiltration	Fatty degeneration/vacuolization
Saline	—	—	—
APAP 400 mg/kg	+++	+++	+++
APA + Carveol 5 mg/kg	+++	++	++
APA + Carveol 10 mg/kg	+	+	+
APA + Carveol 15 mg/kg	—	—	±
APAP + Silymarin 50 mg/kg	—	—	±

+++ significant, ++ high, + moderate, —nil.

#### Effect of Carveol on APAP-Mediated Inflammatory Markers

**Figure 5:**
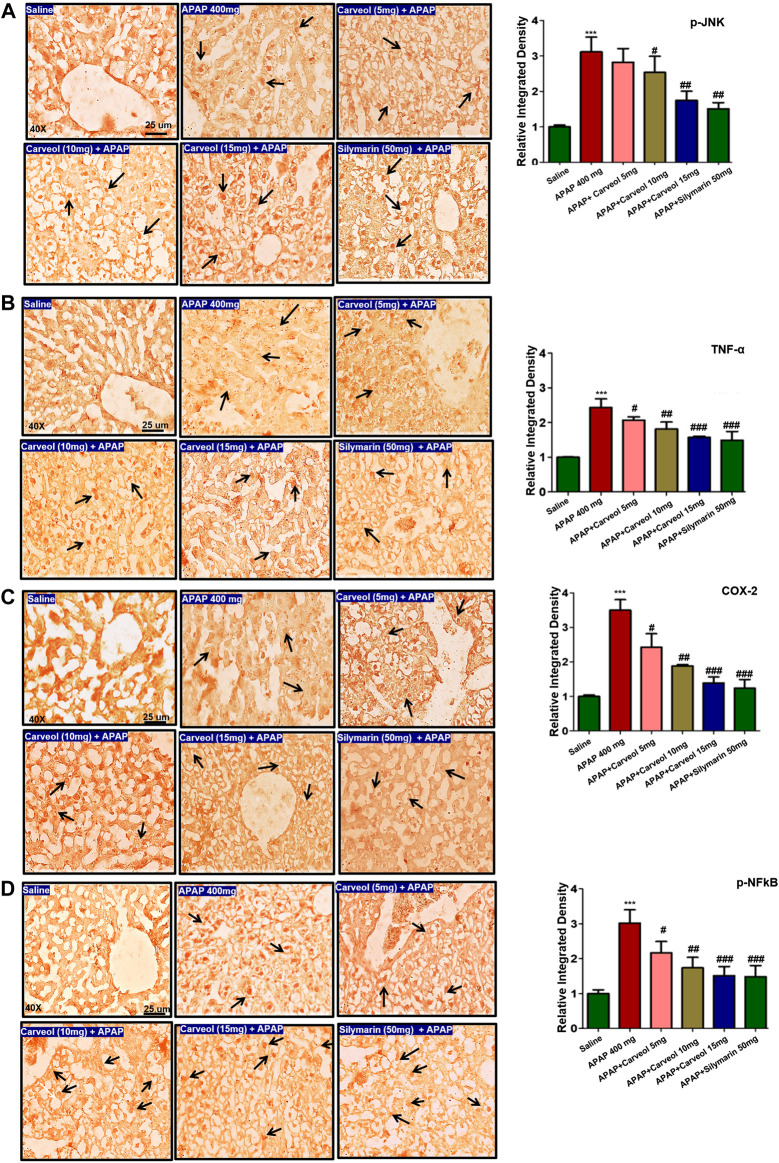
Effect of carveol on inflammatory mediators. The presented images indicate immunoreactivity of **(A)** p-JNK **(B)** TNF-α **(C)** COX-2, and **(D)** p-NFκB. Scale bar = 25 μm, magnification ×40 with *n* = 7/group. The data presented are relative to saline and the number of experiments performed = 3. The data are presented as means ± SEM and were analyzed using one-way ANOVA followed by Tukey’s multiple comparison test. The symbols ∗∗∗ and ### represent significant difference values of *p* < 0.001, while the symbol ## represent *p* < 0.01 values for significant differences and # represents *p* < 0.05. The slides were processed from cohort 1.

The JNK signaling pathway mediates the stress-induced inflammatory cascade and is implicated in mitochondrial apoptosis, and its activation results in the phosphorylation of numerous transcription factors, such as AP-1, p53, Bax, and Bim. Furthermore, JNK can trigger other mediators, such as TNF-α, p-NFκB, and COX-2 (Ali A et al., 2020). To reveal the possible involvement of JNK and TNF-α, immunohistochemical staining was performed, and the results showed higher expression of these mediators in the APAP-administered group (*p* < 0.001) (Figures 5A,B), whereas carveol dose at 15 mg significantly reduced their hyperexpression (*p* < 0.01, Figure 5A, *p* < 0.001, Figure 5B). Moreover, COX-2 and p-NFκB expression were also evaluated, and the results showed that both were highly expressed in the APAP group (*p* < 0.001, Figures 5C,D). Carveol attenuated the expression of p-NFκB and TNF-α in a dose-dependent manner.

#### Effect of Carveol on the Nrf2 Signaling Pathway

**Figure 6:**
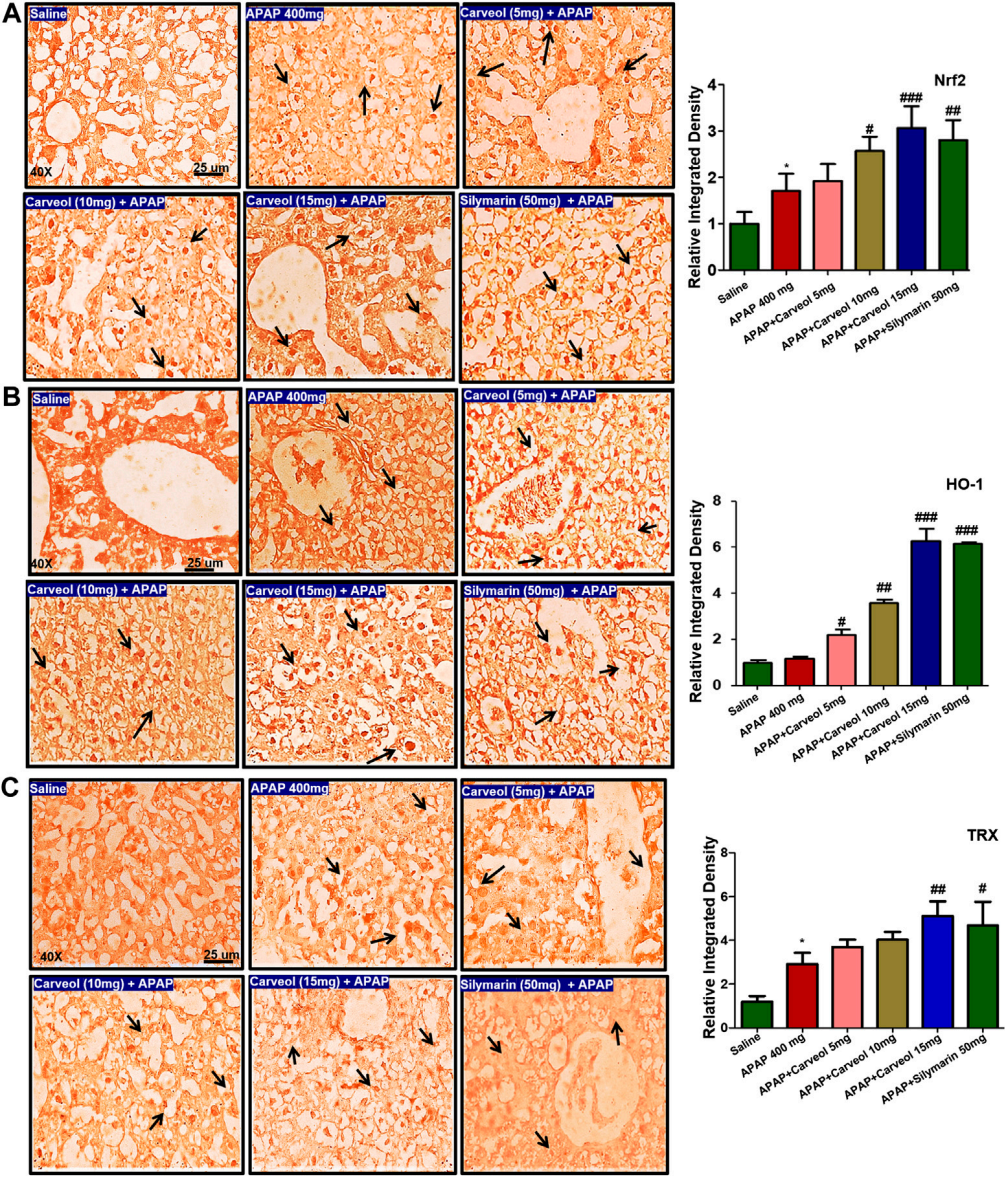
Effect of carveol on immunohistochemistry expression **(A)** Nrf2 **(B)** HO-1, and **(C)** TRX with magnification ×40, and scale bar = 25 μm, *n* = 7/group. Representative histograms indicate a comparatively lower expression of **(A)** Nrf2 **(B)** HO-1, and **(C)** TRX in the APAP group than the carveol group. The data are presented as means ± SEM and were analyzed using one-way ANOVA followed by Tukey’s multiple comparison test. The symbols ### represent significant difference values of *p* < 0.001, while the symbol ## represents *p* < 0.01 values of significant differences, and ∗ or # represents *p* < 0.05. # is significantly different from APAP.

To examine the possible effect of carveol on the Nrf2 signaling pathway, the expression of Nrf2, HO-1, and TRX was determined via immunohistochemistry (Figure 6). APAP activated the expression of Nrf2 (Figure 6A) and TRX (*p* < 0.05, Figure 6C) due to oxidative stress, and carveol, at 15 mg/kg, further expressed these antioxidative proteins to counteract oxidative stress (*p* < 0.001, Figure 6A, *p* < 0.01, Figure 6C). HO-1 and thioredoxin TRX exhibit similar characteristics, as both are antioxidants and eradicate reactive oxygen species, thereby protecting the cell from inflammation and apoptosis (Shah et al., 2018; Li and Liu, 2019). The effect of carveol on the thioredoxin protein level was evaluated in different experimental groups. Representative images and densitometric analysis are shown in Figure 6.

### Results of Experimental Design 2

#### Carveol Enhances the Antioxidant Capacity of the Liver via the Nrf2 Signaling Pathway

**Figure 7:**
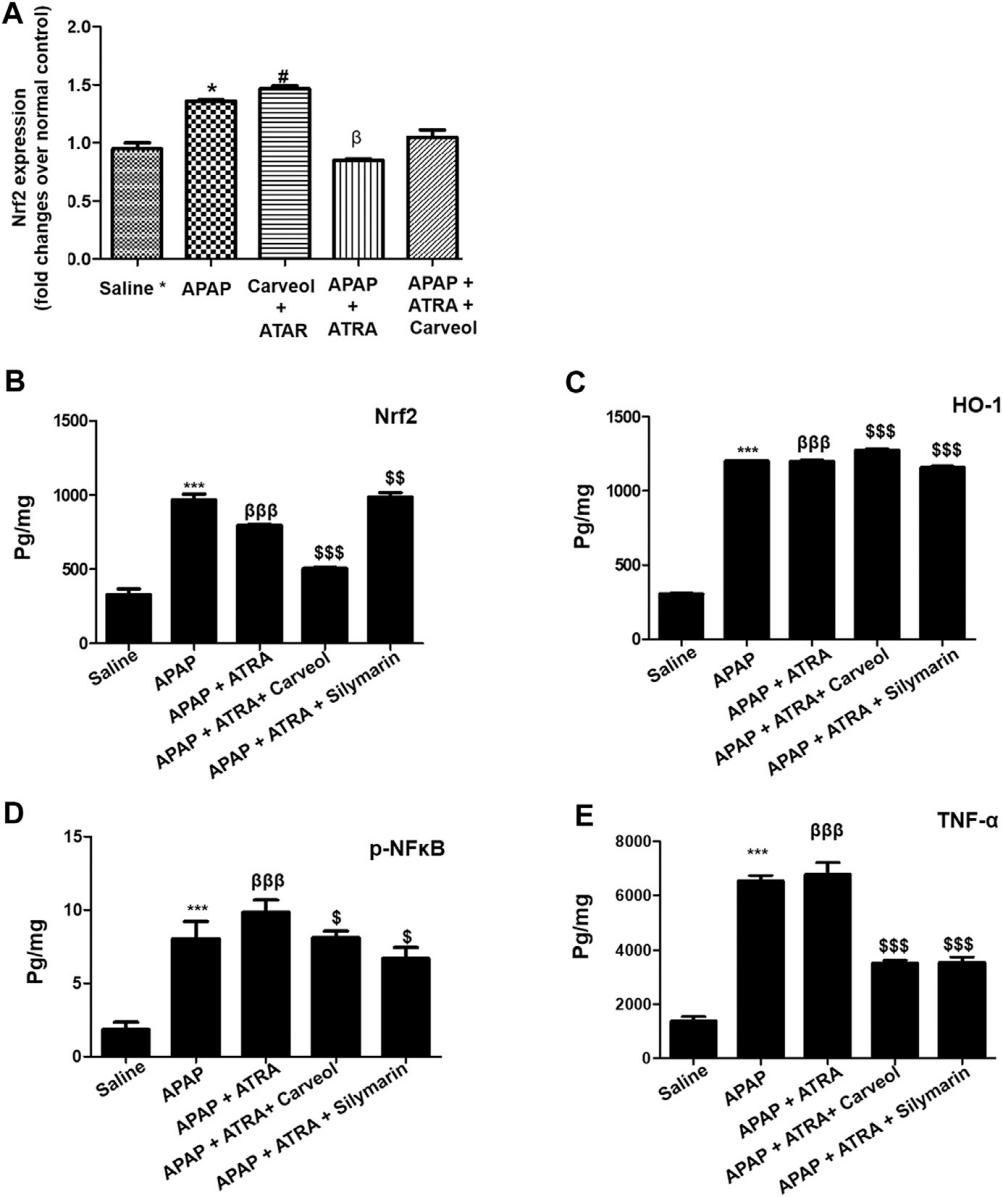
Carveol produces Nrf2-dependent effects. **(A)** qPCR analysis, **(B)** Nrf2 **(C)** HO-1, **(D)** p-NFκB, and **(E)** TNF-α were quantified using ELISA. The data are expressed as mean ± SEM and were analyzed using one-way ANOVA followed by Tukey’s multiple comparison test, and *n* = 5/group. The symbols ∗∗∗, βββ and represent significant difference values of *p* < 0.001, while the symbol $ represents significant difference values of *p* < 0.05. The symbol ∗ or β represents a significant difference relative to saline, while $ represents a significant difference relative to the APAP + ATRA group. The samples were collected 6 h later, for biochemical and morphological analyses. The samples were processed from cohort 2.

**Figure 8:**
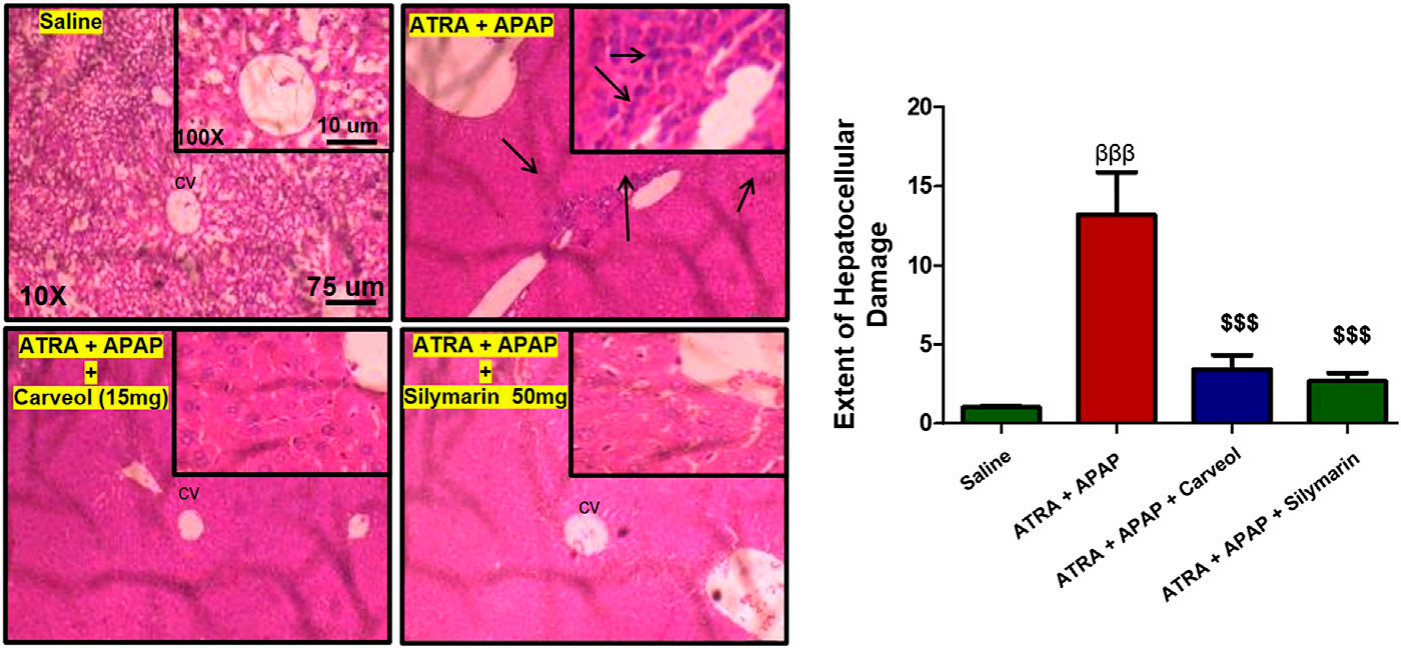
Histological examination and morphological changes in liver tissues. Liver tissues stained with H and E (magnification ×10 and 40×) (*n* = 5/group). The necrotic cells are marked and shown by an arrow; abundant inflammatory cell infiltration can be seen in the APAP + ATRA group. The data are presented as means ± SEM and were analyzed using one-way ANOVA followed by Tukey’s multiple comparison test. The symbols βββ and $$$ represent significant difference values of *p* < 0.001, the symbol β shows a significant difference relative to the saline, and the symbol $ shows a significant difference relative to the APAP + ATRA group. The slides were processed from cohort 2.

To further investigate whether the antioxidative effects of carveol against APAP-induced liver injury, *in vivo*, are Nrf2-dependent, we blocked the Nrf2 effect by using ATRA at a dose of 10 mg/kg. As shown in Figure 7A, Nrf2 gene expression was elevated by carveol at 15 mg dose, while ATRA downregulated this expression. To further validate the hepatoprotective effect, we performed ELISA and we demonstrated similar results for Nrf2 (Figure 7B). The level of HO-1 was also decreased by ATRA (*p* < 0.001). Treatment with carveol at 15 mg/kg modulated the level of Nrf2 and HO-1, whereas ATRA treatment blocked the effects of carveol on Nrf2 (*p* < 0.05, Figures 7B,C). The expression of p-NFκB and TNF-α was also evaluated in these groups, and the results coincided with the Nrf2 findings, with hyperexpression in the ATRA + APAP group (*p* < 0.001). Moreover, carveol (15 mg/kg) attenuated the expression of p-NFκB and TNF-α in the carveol + ATRA + APAP group (*p* < 0.05, Figure 7D, *p* < 0.001, Figure 7E). The results were further validated using biochemical analysis (Tables 4) and H and E staining (Figure 8), with amassing of inflammatory cell migration observed in the APAP + ATRA group (*p* < 0.001, Supplementary Image S1).

**TABLE 4:** ATRA abrogated the effects of carveol.

Treatment	ALT (U/L)	AST (U/L)	TP (g/dl)	ALP (U/L)	LDH (mg/dl)
Saline	67 ± 5.4	89 ± 5.32	6.8 ± 1.56	110 ± 2.7	441.33 ± 14.46
APAP 400 mg/kg + ATRA 10 mg/kg	456 ± 13.25^βββ^	634 ± 16.35^βββ^	2.6 ± 1.7^ββ^	545 ± 9.89^βββ^	3653 ± 4.03^βββ^
APAP + ATRA + Carveol 15 mg/kg	219 ± 12.56^$$$^	376 ± 3.45^$$$^	5.3 ± 1.21^$$^	278 ± 7.89^$$$^	2162 ± 21.72^$$^
APAP + ATRA + Silymarin 50 mg/kg	183 ± 14.23^$$$^	312 ± 8.64^$$$^	5.8 ± 2.1^$$^	267 ± 5.78^$$$^	2017.66 ± 25.91^$$^

ATRA augmented the serum level of LFTs and attenuated the TP, while carveol significantly reduced LFT levels compared to those in the ATRA + APAP group, and increased the total protein level. The symbols βββ and $$$ represent significant difference values of p < 0.001, while the symbol $$ represents p < 0.01 values of significant differences, the symbol $ represents a significant difference relative to APAP + ATRA, while β represents a significant difference relative to the saline group. The data are expressed as means ± SEM and were analyzed using one-way ANOVA followed by Tukey’s multiple comparison test, n = 5. The samples were processed from cohort 2.

#### Docking Studies

**Figure 9:**
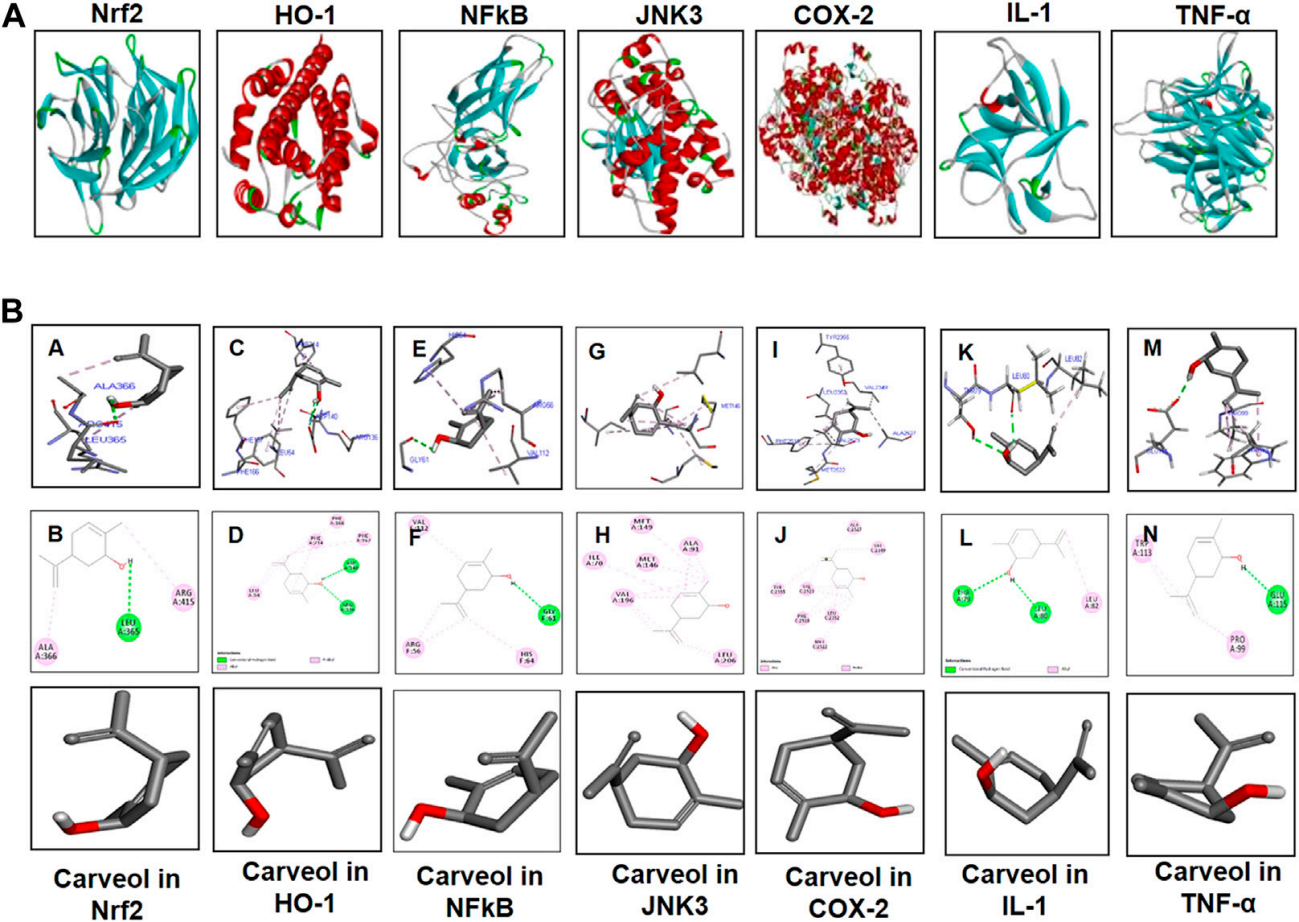
Computational and docking analyses; **(A)** Tertiary structures of the proteins Nrf2, HO-1, NFκB, JNK3, COX-2, TNF-α, and IL-1β. **(B)** The docking results show the best pose of carveol that fitted to Nrf2, HO-1, NFκB, JNK3, COX-2, TNF-α, and IL-1β. The post-docking analysis was visualized using DSV in both 2D and 3D poses. The interaction between carveol and Nrf2 is shown in the panels **(A, B)**, HO-1 in the panels **(C, D)**, NFκB in the panels **(E, F)**, JNK in panels **(G, H)**, COX-2 in the panels **(I, J)**, IL-1 in the panels **(K, L)**, and TNF-α in the panels **(M, N)**. The 3D poses are shown in the panels **(A, C, E, G, I, K, M)** and the 2D in the panels **(B, D, F, H, J, L, N)**. Abbreviations: iNOS, inducible nitric oxide; TNF-α, tumor necrosis factor; IL-1β, interleukin; HO-1, heme oxygenase 1, COX-2, cyclooxygenase.

Comprehensive docking studies were conducted to explore the possible targets of carveol. Cis-carveol was docked in the active catalytic pocket of COX-2, HO-1, IL-1, NFκB, inducible nitric oxide (iNOS), Nrf2, and TNF-α. Table 5 shows the binding energies after docking analysis, and Figure 9 shows the best pose of cis-carveol fitting to COX-2, HO-1, IL-1, NFκB, iNOS, Nrf2, and TNF-α after docking studies. The active sites of these proteins were retrieved from the literature. It was perceived that the hydroxyl group (OH-) of carveol participated in hydrogen bond formation with a protein molecule (Figure 9). The OH- groups of carveol, in these interactions, acted as hydrogen bond donors and the respective protein molecules were hydrogen bond acceptors. ASP-140 and ARG-136 of HO-1, LEU-80, and THR-79 of IL-1β, GLY 61 of NFκB, LEU-365 of Nrf2, and GLU-115 of TNF-α were involved in hydrogen bond interactions. In addition, non-covalent alkyl and Pi-alkyl interactions were observed, which are crucial for temporary interactions, specifically for the drug activity to be proficient in a system.

**TABLE 5:** Binding energy values.

Groups	Hepatocytes necrosis	Inflammatory cells infiltration	Fatty degeneration/vacuolization
Saline	—	—	—
APAP 400 mg/kg + ATRA 10 mg/kg	+++	+++	++
APAP + ATRA + Carveol 15 mg/kg	—	—	—
APAP + ATRA + Silymarin 50 mg/kg	—	—	—

LEU, Leucine; ASP, Aspartate; ARG, Arginine; GLY, Glycine; GLU, Glutamic acid.

## Discussion

**Figure 10:**
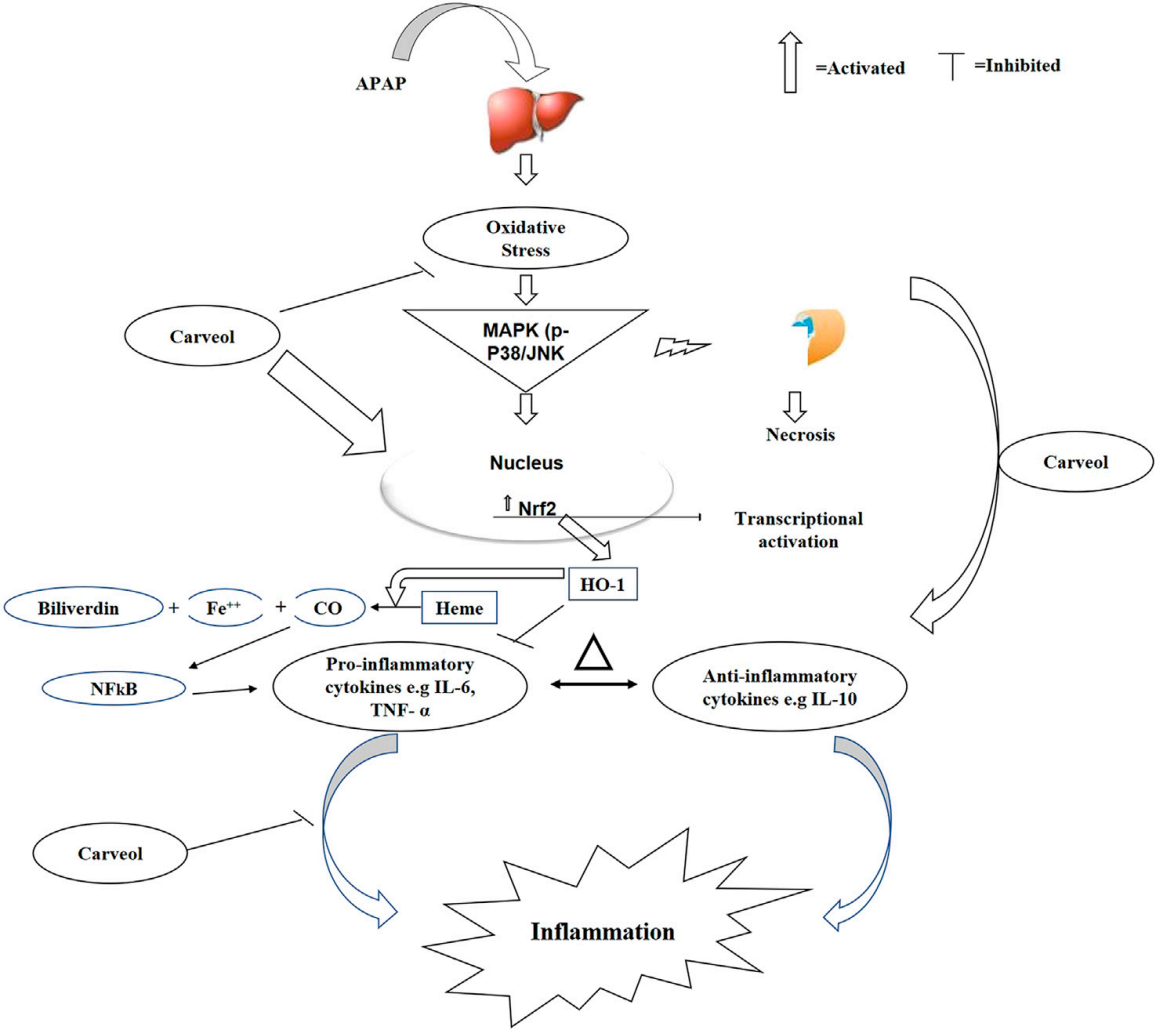
The graphical representation indicates and elaborates the underlying antioxidant and anti-inflammatory mechanisms of carveol against the APAP-induced liver toxicity.

The clinical syndrome of a higher dose of APAP has long been established, and the potent natural antioxidant carveol attenuates APAP-induced detrimental outcomes in hepatic tissue; thus, this study further attested to our previously published data. We previously demonstrated that carveol treatment attenuated ischemic stroke-induced neurodegeneration, by positively affecting the Nrf2 pathway, thereby leading to a reduced infarction area (Malik et al., 2020). We demonstrated here that carveol reversed the oxidative and inflammatory cascades of APAP, possibly by triggering the Nrf2-dependent antioxidative mechanism, which is cross-linked to the pro-survival pathways (Figure 10). Moreover, the low energy values and relatively higher hydrogen bond formation further enhanced complex stability, as revealed through the molecular docking analysis. Additionally, previous studies have reported that targeting inflammation and oxidative stress-coupled targets could provide better therapeutic outcomes (Ali T et al., 2020; Ling et al., 2020), which opens several avenues for the use of natural drug substances.

Paracetamol is an established inducer of liver toxicity in laboratory animals, but the exact pathological mechanism for this toxicity is not well known, and several mechanisms have been proposed. NAPQI, which is a highly reactive metabolite, is mostly attributed to this effect owing to its electrophilic nature, and it attacks several macromolecular targets (Shah et al., 2014; Sharma et al., 2016; Hellerbrand et al., 2017). The reactivity of NAPQI can be abolished by endogenous antioxidant enzymes, such as glutathione (James et al., 2003; Pastore et al., 2003; Lee et al., 2006). We observed a dose-dependent effect. Interestingly, carveol, at both doses, attenuated AST, ALT, and lactate dehydrogenase, along with favorable histological findings.

We have shown here that carveol treatment stimulates Nrf2, a key protein that combats various reactive oxygen species and other stress kinases, thereby halting necrotic and apoptotic cell death in the liver. The implications of inflammation, oxidative stress, and antioxidative mechanisms have been established previously. Our protein analysis using ELISA demonstrated that carveol alleviated the expression of different inflammatory mediators and cytokines, such as TNF-α and NFκB, which is further linked to increasing Nrf2 nuclear translocation and activation of the antioxidant machinery. Furthermore, such cascading events diminished the release of pro-inflammatory mediators and cytokines, by downregulating the NFκB signaling pathway, similar to previously reported data (Ali A et al., 2020). Nrf2 played a pivotal role in our model, as it reciprocally modulated the oxidative stress-induced inflammation. This notion was further demonstrated when ATRA treatment wore off the hepatoprotective effects of carveol and elevated the expression of the inflammatory markers. These results are consistent with other experimental models, in which Nrf2 shields against inflammation (Mohsin Alvi et al., 2020). Thus, its activation, either pharmacological or signaling cascades, rescues the tissue from the hallmarks of inflammation and oxidative stress, while its pharmacological inactivation deteriorates the pathological conditions in several other related degenerative models. Furthermore, the downstream targets of Nrf2, such as HO-1 and TRX, later mediated the protective mechanism of Nrf2. It is worth mentioning here that natural drugs are frequently reported to activate the cleavage of Nrf2-Keap1 dimer and thus allow the translocation of Nrf2 to the nucleus, to stimulate antioxidant machinery, including HO-1 and NAPDH quinine dehydrogenase-1 (NQO1), and thus provide a notable antioxidative mechanism to reverse the oxidative stress-induced inflammation (Jaiswal, 2004).

GSH, SOD, and CAT are among the first-line defense antioxidants that are important and indispensable in the defense of oxidants, particularly in the liver. SOD catalyzes the conversion of superoxide free radicals to H_2_O_2_ and O_2_, while CAT helps in protecting against the harmful effects of superoxide and lipid peroxidation in the liver. Our results demonstrated that carveol significantly attenuated hepatic MDA, a biomarker of lipid peroxidation, and leveled the GSH, SOD, and CAT contents, indicating a strong and complex effect of carveol in alleviating the APAP-induced oxidative stress. GSH activity is vital both for sustaining cellular homeostasis and for eliminating free radicals, such as superoxide. Furthermore, consistent studies have reported the detoxifying effect of GSH against electrophiles, such as NAPQI (Zai et al., 2018). Moreover, several protective agents act against liver insults, by normalizing GSH content. Thus, increased GSH, SOD, and CAT biosyntheses could account for the underlying mechanism of carveol against the NAPQI-induced oxidative stress and inflammation.

The Nrf2 pathway has a prominent protective role in the liver, while APAP exerts a detrimental effect on the Nrf2 pathway. A higher level of toxicity was observed in Nrf2-null mice than in wild-type mice when exposed to hepatotoxic agents (Aleksunes et al., 2008; Patterson et al., 2013; Guo and White, 2016). Previous studies on traditional Chinese drugs have shown their ability to activate Nrf2 and protect against the APAP-induced liver injury in mice. Consistent literature suggests that activation of the antioxidant machinery, such as Nrf2, could downregulate oxidative stress and the inflammatory cascade machinery, such as the NFκB pathway and cytokines (TNF-α and COX-2) (Ali A et al., 2020). The critical role of carveol in mediating the antioxidative effect of Nrf2 was further validated with the Nrf2 antagonist ATRA (Mohsin Alvi et al., 2020). ATRA administration removed the hepatoprotective effect of carveol, abolished the increased levels of Nrf2 and HO-1, and further exaggerated p-NFκB and TNF-α levels.

Several studies have documented the cross-talk between oxidative stress and the inflammation process (Al Kury et al., 2019; Al Kury et al., 2020). Therefore, drug therapeutics should be designed to subside the inflammatory process and oxidative distress, by triggering the endogenous antioxidant defense system. We also studied the expression profile of a thiol-related protein, thioredoxin (TRX), an integral enzyme in hemostatic redox reactions (Patterson et al., 2013). Carveol treatment boosted the TRX level, which further validated the antioxidant nature of carveol in APAP-induced liver injury. However, the exact mechanism of how carveol abrogated liver injury needs to be explored in detail.

APAP provokes free radical formation and subsequent pro-inflammatory mediators. Moreover, role of the JNK pathway in inflammatory cascades and cellular death has been strongly established (Ali A et al., 2020), and ROS and inflammatory cytokines can trigger JNK activation (Ali A et al., 2020; Ali T et al., 2020; Ling et al., 2020). Furthermore, the p-JNK pathway has been implicated in various animal models and human diseases (Shah et al., 2018; Li and Liu, 2019; Ali A et al., 2020; Ling et al., 2020; Malik et al., 2020), and the downregulation or inhibition of this pathway has been found to contribute to the protective strategy (Shah et al., 2019). We observed similar activities in the APAP-intoxicated group, and carveol significantly reduced p-JNK expression. Furthermore, hepatic necrosis can be attenuated by downregulating p-NFκB expression, which may further act on the downstream COX-2 and iNOS and thus reduce ROS generation. Moreover, the expression of COX-2 and iNOS can be prevented by antagonizing p-NFκB expression (Ali A et al., 2020). Natural drug substances have significant anti-inflammatory potential, and we previously showed the inhibitory effect of carveol, polydatin, and Ginkgo biloba on NFkB, COX-2, and iNOS expression in different experimental models (Al Kury et al., 2019; Al Kury et al., 2020; Malik et al., 2020). We postulated here that carveol reduces hepatocellular necrosis by negatively modulating the expression of mitogen kinase and other inflammatory cytokines.

We performed docking analysis using the Autodock Vina program. The binding energy was evaluated for carveol and the respective proteins (Figure 9; Table 5). Different intermolecular interactive forces are vital for energetically stabilizing the drug-receptor complex. Carveol is flexibly complexed with protein targets, by establishing H-bonds and other hydrophobic interactions. Hydrogen bond formation is important for stabilization, recognition, and molecular movement (Baker and Hubbard, 1984; Desiraju and Steiner, 2001). Several studies have revealed the importance of this kind of bonding in ligand-protein complex stability, at a bond distance of 2.6Ao–3.2Ao (Glusker, 1995; Sarkhel and Desiraju, 2004; Panigrahi and Desiraju, 2007). We speculate that the formation of H-bonds between the ligand carveol and the respective protein supports the corresponding complex stability.

## Conclusion

In summary, our *in vivo* results demonstrate that carveol could be a potent antioxidant and anti-inflammatory agent that mediates protective properties in APAP-induced liver toxicity. Furthermore, our proposed mechanism suggests that carveol may activate the master endogenous antioxidant protein Nrf2 and may be associated with the negative modulation of p-JNK and other neuroinflammatory mediators; it may, thus, offer a new therapeutic option for preventing and managing oxidative stress and inflammation in degenerative disorders.

## Ethics Statement

The animal study was reviewed and approved by Research and Ethical Committee of Riphah institute of Pharmaceutical Sciences, Riphah International University Islamabad Pakistan.

## Author Contributions

All authors made substantial contributions to conception and design, acquisition of data, or analysis and interpretation of data; took part in drafting the article or revising it critically for important intellectual content; agreed to submit to the current journal; gave final approval of the version to be published, and agree to be accountable for all aspects of the work.

## Funding

This work was supported by the Natural Science Foundation of Shenzhen University General Hospital Grant No: SUGH2019QD018, and the Natural Science Foundation of Shenzhen University General Hospital Grant No: SUGH2020QD015.

## Conflict of Interest

The authors declare that the research was conducted in the absence of any commercial or financial relationships that could be construed as a potential conflict of interest.
