# Supplementary figures and images for: Carveol a Naturally-Derived Potent and Emerging Nrf2 Activator Protects Against Acetaminophen-Induced Hepatotoxicity
Source: Front Pharmacol. 2021 Jan 28;11:621538. doi: 10.3389/fphar.2020.621538 (PMC7883019; doi:10.3389/fphar.2020.621538)

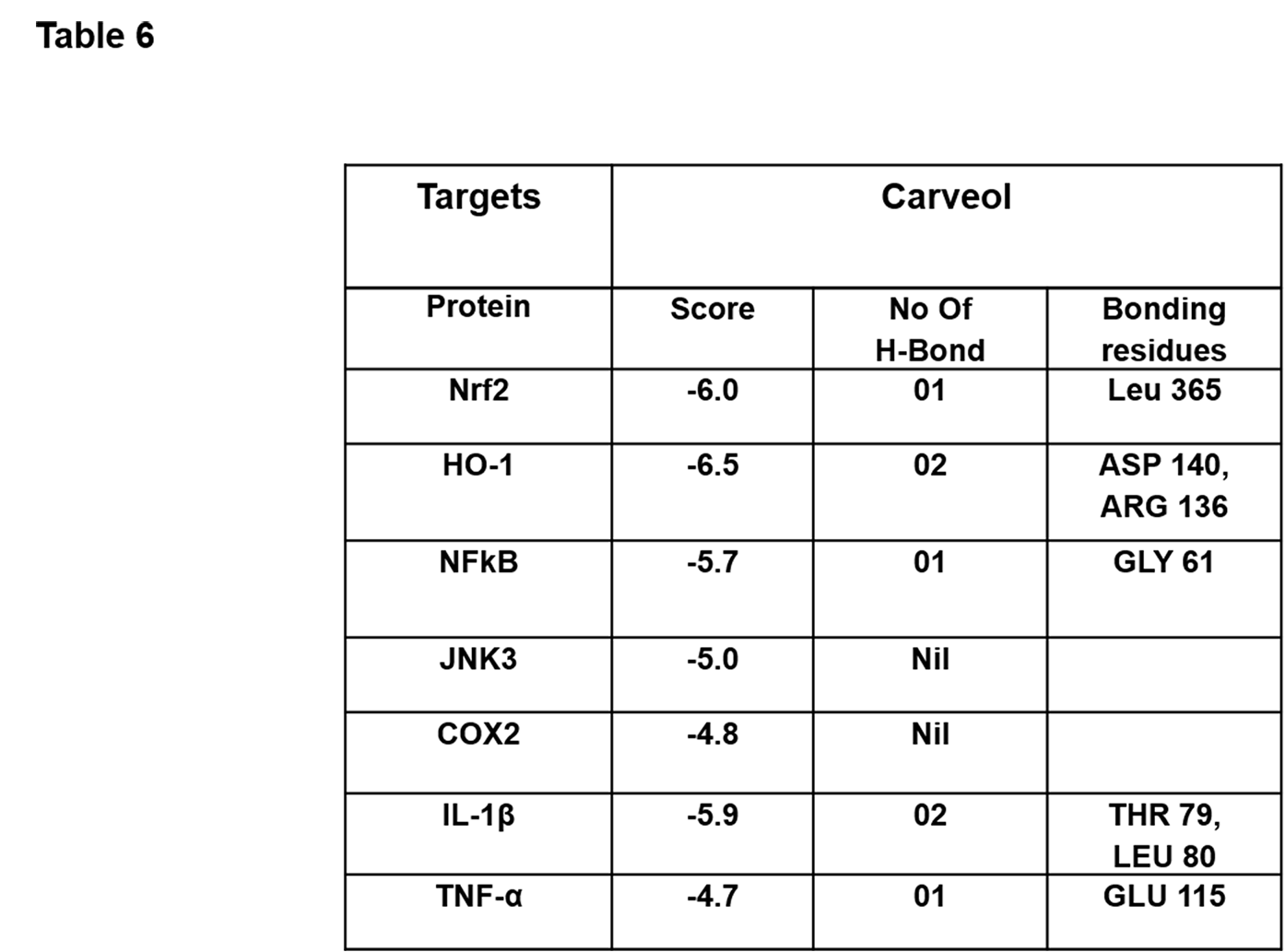

Supplement: Supplementary file 1 [file image1.tif]
